# The developmental and structural uniqueness of the embryo of the extremophile viviparous nematode, *Tokorhabditis tufae*

**DOI:** 10.3389/fphys.2023.1197477

**Published:** 2023-06-22

**Authors:** Tatsuya Yamashita, Taisuke Ekino, Natsumi Kanzaki, Ryoji Shinya

**Affiliations:** ^1^ School of Agriculture, Meiji University, Kawasaki, Japan; ^2^ Kansai Research Center, Forestry and Forest Products Research Institute (FFPRI), Kyoto, Japan

**Keywords:** viviparity, embryonic development, provisioning nutrients, evolution, extremophile

## Abstract

Viviparity, a reproductive form that supplies nutrients to the embryo during gestation, has repeatedly and independently occurred in multiple lineages of animals. During the convergent evolution of viviparity, various modifications of development, structure, and physiology emerged. A new species of nematode, *Tokorhabditis tufae*, was discovered in the alkaline, hypersaline, and arsenic-rich environment of Mono lake. Its reproductive form is viviparity because it is obligately live-bearing and the embryo increases in size during development. However, the magnitude of the increase in size and nutrient provisioning are unclear. We measured egg and embryo sizes at three developmental stages in *T*. *tufae*. Eggs and embryos of *T*. *tufae* at the threefold stage were respectively 2.6- and 3.6-fold larger than at the single-cell stage. We then obtained *T. tufae* embryos at the single-cell, lima bean, and threefold developmental stages and investigated the egg hatching frequency at three different concentrations of egg salt buffer. Removal of embryos from the uterus halted embryonic development at the single-cell and lima bean stages in *T. tufae* irrespective of the solution used for incubation, indicating the provision of nutrients within the uterus. Ultrastructural and permeability evaluation showed that the permeability barrier did not form during embryonic development, resulting in increased molecular permeability. This high permeability caused by the absence of the permeability barrier likely enables supply of nutrients from the mother. The structural and physiological modifications in *T*. *tufae* are like those in other viviparous animals. We conclude that *T*. *tufae* is a viviparous rather than an ovoviviparous nematode. *T*. *tufae* will facilitate investigation of the evolution of viviparity in animals.

## Introduction

Viviparity is a form of reproduction in which embryonic development is supported by nutrients in the body of the mother and has evolved multiple times independently in multiple lineages of animals (reviewed in Blackburn, 2015; Ostrovsky et al., 2016). This suggests a convergent evolution event along the animal phylogenetic tree, likely in response to similar selective forces driving similar adaptations. Several environmental factors driving the evolution of viviparity have been assumed in animals (Hogarth, 1976). The best-studied viviparous animals for understanding the evolution of viviparity are squamate reptiles (Blackburn, 1998; Van Dyke et al., 2014). In squamate reptiles, the driving force is believed to be cold climate. This hypothesis is underpinned by the habitat of viviparous species and phylogenetic analyses indicating that most resent transitions occurred in cold climates (Hodges, 2004; Lambert and Wiens, 2013; Watson et al., 2014). However, whether viviparity arose from a common reproductive system and its driving force in animal lineages are unclear.

Nematodes are excellent model organisms for studying viviparity evolution, given their ecological diversity and ease of genetic manipulation with a short life cycle. Most nematodes are oviparous: females/hermaphrodites lay eggs, which hatch into the external environment. Oviparous mothers retain eggs in the uterus under some stressful conditions. The larvae hatched in the uterus consume the body contents of the mother, and emerge through the mother’s body wall, generally resulting in the mother’s death. This facultative vivipary is known as “bagging” (also known as *endotokia matricida*) (Lordello, 1951; Johnigk and Ehlers, 1999; Chen and Caswell-Chen, 2004). In contrast, females/hermaphrodites of some nematode species obligately retain fertilized eggs within their reproductive track and give birth to larva; these are described as ovoviviparous or viviparous. Here, we define ovoviviparity as birth following intrauterine hatching from an egg from a rigid eggshell without embryonic growth, whereas viviparous animals gestate the embryo in the uterus until the embryos become larvae (Balinsky, 1970). Since nutrition is supplied by the mother in viviparous animals, the embryo typically does not have a rigid eggshell in viviparous animals, such as insects (Tworzydlo et al., 2013), squamate reptiles (Blackburn, 1993a; Blackburn, 1993b; Blackburn, 1998), and sharks (Lombardi and Files, 1993; Heiden et al., 2005). Some nematode species have been reported to be viviparous nematodes, *e.g*., *Trichinella spiralis*, filarial nematodes (Smyth, 1994; Hugot et al., 2001). However, in most cases where these features have been adequately described, this vivipary, which is ovovivipary as defined above, is the retention of eggs, wherein embryonic development proceeds as in oviparous species. In nematodes, no examination of nutrient supply and eggshell structure has made a clear distinction between ovoviviparous and viviparous species. A new species of nematode, *Tokorhabditis tufae*, was discovered in the alkaline, hypersaline, and arsenic-rich environment of Mono Lake, California; being obligately live-bearing, *T*. *tufae* is likely viviparous (Shih et al., 2019; Kanzaki et al., 2021). Kanzaki et al. (2021) observed the embryos with differential interference contrast (DIC) microscopy and showed that *T*. *tufae* embryos increase in size during embryonic development, suggesting nutrient supply from mother to embryo. Although the magnitude of the increase in size and nutrient provisioning are unclear, the form of reproduction in *T. tufae*, based on the definition above, is consistent with viviparity rather than ovoviviparity.

To confirm the viviparity of *T*. *tufae* and to demonstrate its developmental and morphological uniqueness, we measured embryo size at various developmental stages and calculated the growth rate. Subsequently, we investigated the permeability of substances of various molecular weights in *T*. *tufae* and its closely related egg-laying species, *Auanema rhodensis. Auanema* is the sister group of *Tokorhabditis* and shares similar features, such as a trioecious mating system (see Figure 9 in Kanzaki et al., 2021 for their phylogenetic relationship). Given the high substance permeability of *T*. *tufae*, the ultrastructure of the eggshell and permeable barrier was visualized by transmission electron microscopy (TEM).

## Materials and methods

### Nematode culture and maintenance

*T*. *tufae* strain PS8402 was isolated from soil sampled at Mono Lake, CA (Shih et al., 2019). *A*. *rhodensis* strain SB347 was isolated from a deer tick in Rhode Island (Kanzaki et al., 2017b), and has an oviparous reproductive mode. Nematodes were cultured on nematode growth medium (NGM; 3 g NaCl, 2.5 g peptone, 15 g agar, and 975 mL H_2_O; autoclaved and cooled to approximately 55°C; and 1 mL of 1 M CaCl_2_, 1 mL of 5 mg/mL cholesterol in ethanol, 1 mL of 1 M MgSO_4_, and 25 mL of 1 M KPO_4_ buffer added) seeded with *Escherichia coli* OP50 as a food source. Nematodes were maintained at 20°C.

### Collection of gravid young adult hermaphrodites

To assess eggshell structure and permeability, we collected adult hermaphrodites of *T*. *tufae* and *A*. *rhodensis* with fertilized eggs. Because all nematodes of both species recovered from dauer larvae become hermaphrodites, we picked dauer larvae from the culture plates, and incubated them for 30–40 h on NGM seeded with *E*. *coli* OP50. Young adult hermaphrodites were used for subsequent analyses.

### Measurement of embryo size

In a preliminary experiment, fertilized eggs of *T*. *tufae* from hermaphrodites stopped development immediately upon being removed from the hermaphrodite and incubated in egg salt buffer (118 mM NaCl, 40 mM KCl, 3.4 mM CaCl_2_, 3.4 mM MgCl_2_, and 5 mM HEPES). Therefore, we obtained embryos at the single-cell, lima bean, and threefold (pretzel) developmental stages and measured their size. The embryos were collected by dissecting hermaphrodites using a surgical needle (Terumo, NN-2719S) or picked from culture plates using a nickel wire pick and photographed under a DIC microscope (Olympus, BX53) equipped with a camera (Hamamatsu, ORCA-spark). The area occupied by each embryo was measured using ImageJ v. 1.53a (Rasband, 2014; https://imagej.nih.gov/ij/). At least ten biological replicates of each embryonic stage were examined.

### Egg hatching frequency in egg salt buffer

Embryos were collected by dissecting adult hermaphrodites or were picked from culture plates of *T*. *tufae* and *A*. *rhodensis*. We obtained embryos at the single-cell, lima bean, and threefold (pretzel) developmental stages and transferred them to 0.4×, 0.7×, or 1.0× egg salt buffer. The embryos were incubated at 20°C for 2 days and the ratio of the number of hatched larvae per the number of incubated eggs was examined under a stereoscope (Zeiss AxioZoom V16, ZEISS).

### Embryo permeability analysis

Embryos were incubated with Texas Red 3000 MW lysin-fixable dextran (Thermo Fisher Scientific, D3328), Texas Red 10,000 MW neutral dextran (Thermo Fisher Scientific, D1828), and Texas Red 70,000 MW neutral dextran (Thermo Fisher Scientific, D1830). Permeability was analyzed as described by Olson et al. (2012) with small modifications. Dextran solutions were diluted in 0.7× egg salt buffer and adjusted to 1.25 mg/mL. The embryos were incubated in dextran solutions for 30 min in the dark at room temperature. After rinsing in 0.7× egg salt buffer, embryos were imaged under a confocal microscope (ZEISS, LSM 880 with AiryScan).

### Visualization of eggshell ultrastructure

Fertilized eggs and gravid adult hermaphrodites were observed by TEM. The formation of the eggshell and the permeability barrier is completed immediately after fertilization in *Caenorhabditis elegans* (Olson et al., 2012). Here, fertilized eggs (random stage) of *T*. *tufae* and *A*. *rhodensis* were collected by dissecting hermaphrodites using a surgical needle in M9 buffer. Samples for TEM were prepared following the method of Ekino et al. (2017). Eggs or gravid adult hermaphrodites were fixed in 2.5% glutaraldehyde and 2% paraformaldehyde in 0.1 M phosphate buffer (pH 7.4) overnight. Fixed samples were packed in 2% water agar and infiltrated in fixative for 1 h. After rinsing six times for 10 min each in 0.1 M phosphate buffer (pH 7.4), agarose pieces including eggs or adults were post-fixed in 1% osmium tetroxide for 90 min in 0.1 M phosphate buffer (pH 7.4). After rinsing three times for 10 min each in distilled water, samples were dehydrated in a graded ethanol series (50%, 70%, 80%, 90%, and three times in 99.5% in water, 10 min each) and cleaned three times for 10 min each with propylene oxide. Samples were infiltered overnight in a mixture of 50% Eponate resin and 50% propylene oxide and again in undiluted Eponate resin. Finally, they were embedded in Eponate resin. The Eponate resin was prepared according to Kadoya. (2010). Samples were sectioned using a diamond knife (Nisshin EM Co., ultratrim and ultra 45°) in an ultramicrotome (Leica, Ultracut UCT). Sections (70 nm in thickness) were collected on formvar-coated copper grids for electron microscopy. The grids were stained with EM stainer (Nisshin EM Co., 336) for 30 min followed by lead citrate for 5 min. Grid-mounted sections were examined and photographed at 100 kV using TEM (JEOL, JEM-2000EX).

## Results

### *T*. *tufae* embryo size

**FIGURE 1:**
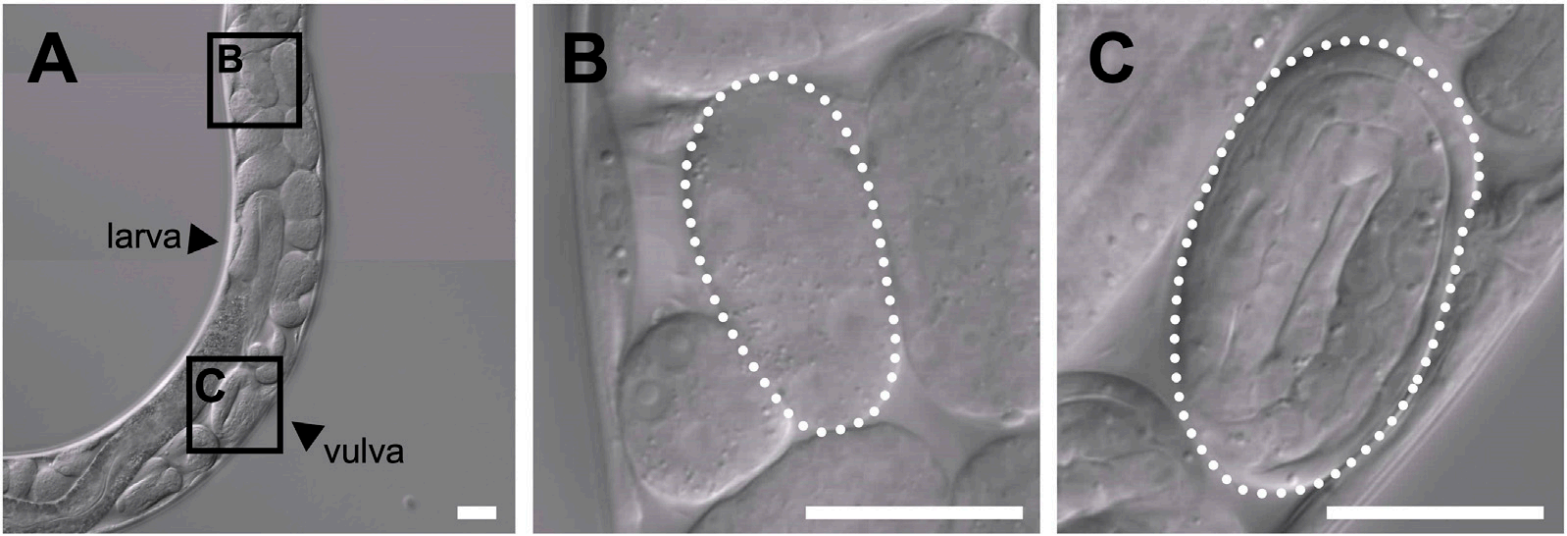
Differential interference contrast images of embryos of a gravid adult hermaphrodite *Tokorhabditis tufae*. **(A)** Reproductive system of a gravid adult hermaphrodite. Two stages of embryos are boxed in **(A)** and shown at higher magnification in **(B)** and **(C)**. **(B)** Early-stage embryo. **(C)** Threefold (pretzel)-stage embryo (scale bar, 20 μm).

**FIGURE 2:**
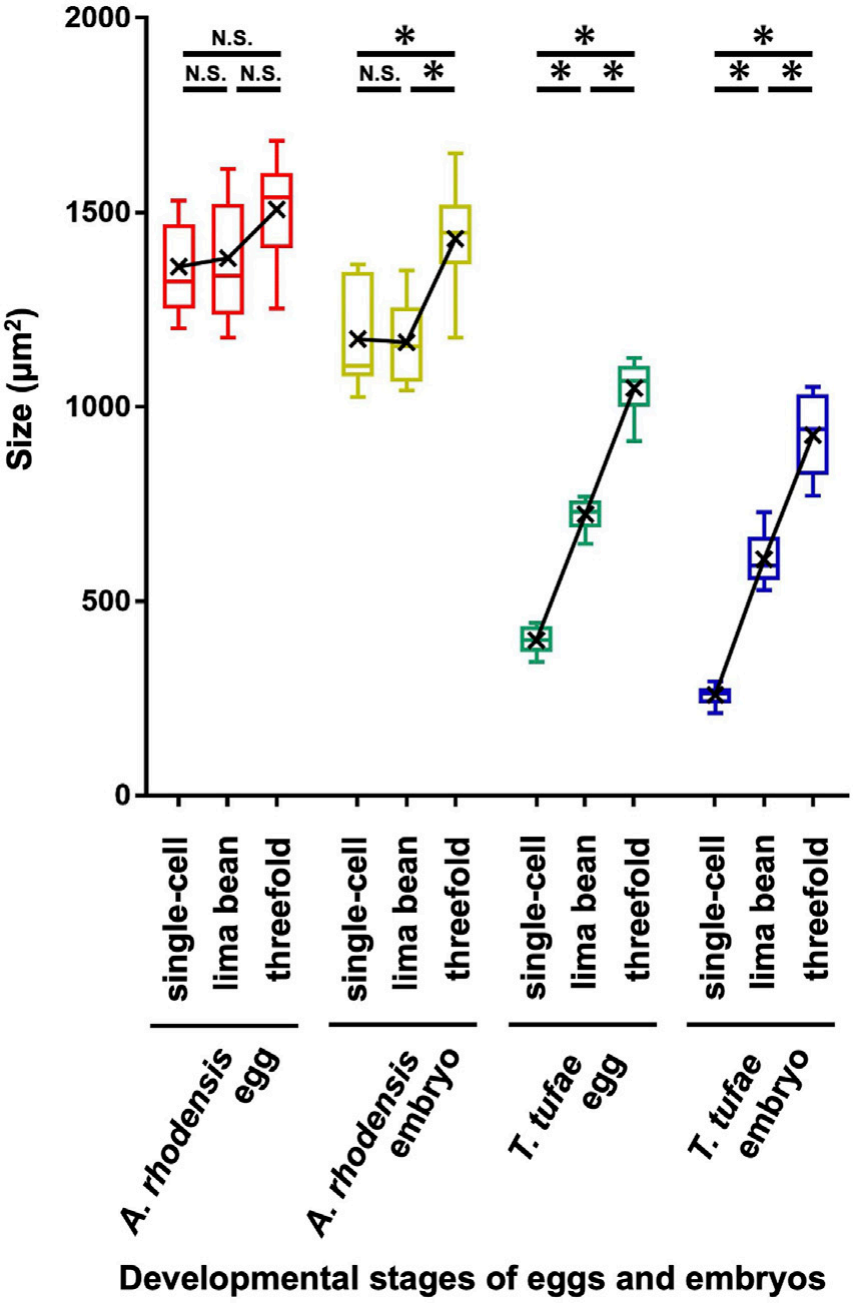
Egg and embryo sizes at the single-cell, lima bean, and threefold stages in *Auanema rhodensis* and *Tokorhabditis tufae*. Medians (-), means (x), and lower and upper quartiles are shown. At least ten biological replicates of embryos per stage were examined. The Tukey–Kramer test was used to compare the size of the egg or embryo (*p < 0.01; N.S. *p* > 0.05).

In the uterus of *T*. *tufae* adult hermaphrodites, we observed embryos at various developmental stages and larvae (Figure 1A) by DIC microscopy. Embryo size differed according to developmental stage (Figures 1B,C). We measured egg and embryo sizes at the single-cell, lima bean, and threefold stages. No significant differences in the egg size of *A. rhodensis* were observed among the three stages, and significant differences in embryo size were observed only between the threefold stage and the other stages (Tukey–Kramer test; *p* < 0.01). In contrast, significant differences in egg and embryo size were observed among all stages of *T. tufae* (Tukey–Kramer test; *p* < 0.01) (Figure 2). Eggs at the threefold stage were approximately 2.6-times larger than at the single-cell stage, compared to approximately 1.1-times for the oviparous *A*. *rhodensis* (Figure 2). Embryos at the threefold stage were approximately 3.6-times larger than at the single-cell stage in *T*. *tufae*, compared to approximately 1.2-times for the oviparous *A*. *rhodensis* (Figure 2).

### Egg hatching frequency in egg salt buffer

We obtained *T. tufae* and *A. rhodensis* embryos at the single-cell, lima bean, and threefold developmental stages and investigated the egg hatching frequency at three different concentrations of egg salt buffer. A certain number of *A. rhodensis* larvae hatched at all three embryonic stages (Table 1). In particular, 71.4% of the embryos hatched in 1.0× egg salt buffer at the single-cell stage. In contrast, when single-cell or lima bean stage *T. tufae* embryos were tested, none subsequently hatched in any of the buffers examined. Significant differences were observed in the hatching frequency of *T. tufae* and *A. rhodensis* at all embryonic stages and in all of the buffers tested (χ^2^ test; degrees of freedom [d.f.] = 1, *p* < 0.0001; Table 1).

**TABLE 1:** Hatching rates of *Auanema rhodensis* and *Tokorhabditis tufae* embryos in egg salt buffer.

		***A. rhodensis***		***T. tufae***		Chi-square value	*p*	
		n	Hatching rate (%)	n	Hatching rate (%)			
Embryo stage	Concentration of ESB							
Single-cell stage	×0.4	37	35.1	31	0.0	31.08	2.48E-08	****
	×0.7	34	50.0	32	0.0	21.55	3.45E-06	****
	×1.0	42	71.4	34	0.0	40.12	2.38E-10	****
Lima bean stage	×0.4	35	45.7	35	0.0	20.74	5.26E-06	****
	×0.7	51	41.2	46	0.0	24.17	8.80E-07	****
	×1.0	35	74.3	34	0.0	40.53	1.94E-06	****
Threefold stage	×0.4	45	57.8	51	2.0	36.84	1.28E-09	****
	×0.7	42	64.3	31	9.7	21.97	2.77E-06	****
	×1.0	66	81.8	38	0.0	64.67	8.86E-16	****

The hatching rate was calculated as the number of hatched larvae divided by the total number of embryos collected. The chi-square test was used to compare the hatching rates between *Auanema rhodensis* and *T. tufae* at the same embryonic stage and at the same concentration of egg salt buffer (ESB) (d.f. = 1, ****p < 0.0001).

### Egg permeability

**FIGURE 3:**
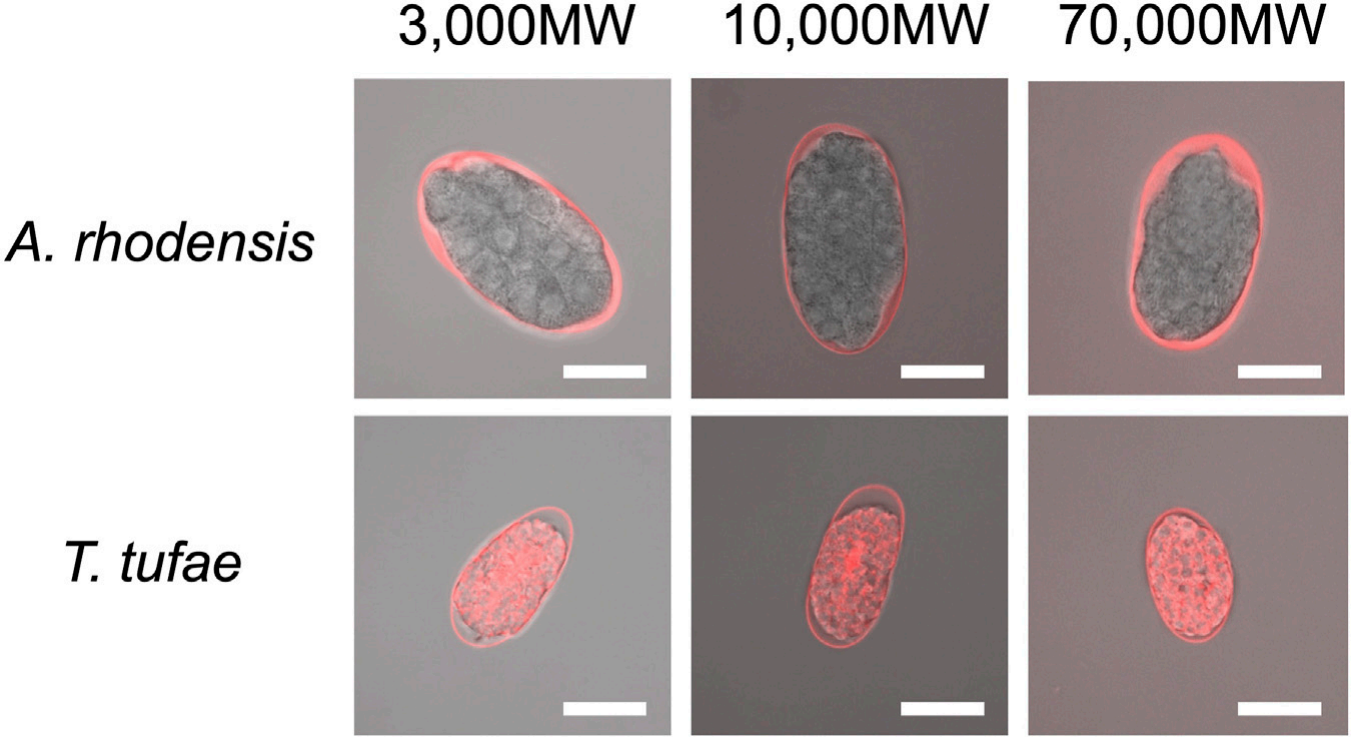
Confocal micrographs of eggs and embryos of *Tokorhabditis tufae* and *Auanema rhodensis* after incubation with fluorescent substances of 3,000, 10,000, and 70,000 MW (scale bar, 20 μm).

We incubated eggs in solutions of fluorescent substances of a variety of molecular sizes. In *A*. *rhodensis*, we observed fluorescence only in the region between the eggshell and the embryo irrespective of substance molecular size (Figure 3). All the fluorescent substances permeated *T*. *tufae* embryos (Figure 3).

### Eggshell ultrastructure in *T*. *tufae* and *Auanema rhodensis*

**FIGURE 4:**
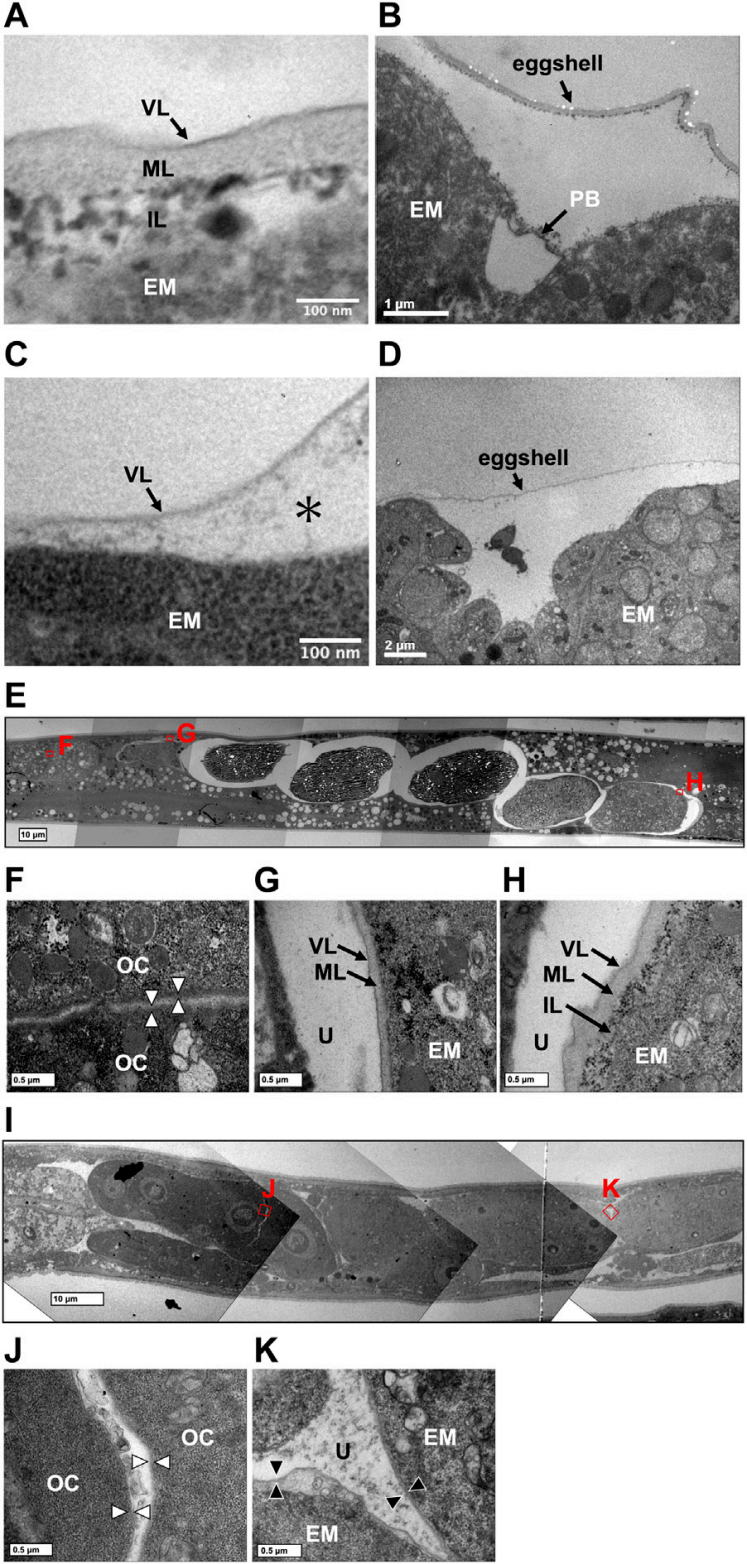
Transmission electron micrographs of eggshells and permeability barriers in *Tokorhabditis tufae* and *Auanema rhodensis*. Eggshells of **(A)** *rhodensis* **(A)** and *T*. *tufae* **(C)** (* fluid-filled layer, perimembrane space). The permeability barrier was formed between the eggshell and the embryo in **(A)** *rhodensis* **(B)**. No permeability barrier in *T*. *tufae* **(D)**. **(E–H)** Transition of the ultrastructure surrounding the embryo in **(A)** *rhodensis* (F, oocyte; G, embryo during eggshell formation; H, embryo after eggshell formation). **(I–K)** Transition of ultrastructure surrounding the embryo in *T*. *tufae* (J, oocyte; K, fertilized egg). EM, embryo; IL, inner layer; ML, middle layer; OC, oocyte; PB, permeability barrier; U, uterus; VL, vitelline layer.

A rigid eggshell often disappears in viviparous animals (Blackburn, 1993a; Blackburn, 1993b; Heiden et al., 2005). We visualized the ultrastructure of *T*. *tufae* and *A*. *rhodensis* eggshells by TEM. The eggshell of *A*. *rhodensis* was composed of a vitelline layer (VL), middle layer (ML), and inner layer (IL), and there was a permeable barrier between the eggshell and embryo (Figures 4A,B). This structure is like that of another oviparous species, *C*. *elegans* (Stein and Golden, 2018). However, the *T*. *tufae* eggshell consisted of only a single layer (Figure 4C). In addition, a permeable barrier was absent in *T*. *tufae* eggs (Figure 4D); the density of staining suggested this to be a VL.

We next visualized the plasma membrane of oocytes and the eggshell of fertilized eggs in the uterus of hermaphrodites (Figures 4E–K). In *A*. *rhodensis*, no eggshell was observed in oocytes in the proximal gonad (Figure 4F). Eggs immediately after sperm entry/fertilization had a VL, which is indistinguishable from the oocyte membrane, and a pale ML inside the VL (Figure 4G). In fertilized eggs in the uterus, a dark IL was present inside the ML (Figure 4H). In *T*. *tufae*, the structures of the outer layers of oocytes (Figure 4J) and fertilized eggs (Figure 4K) were indistinguishable. Fertilized *T*. *tufae* eggs lacked a ML and IL, indicating disappearance of a rigid eggshell.

## Discussion

We have reported that *T*. *tufae* has a viviparous reproductive mode because embryos increase in size during embryonic development. However, we were concerned that the embryos were deformed due to spatial constraints in the gonad and uterus. Here, we measured egg and embryo sizes (area) at three developmental stages in *T*. *tufae* and *A*. *rhodensis*. Single-cell-stage embryos of *T*. *tufae* were smaller than those of *A*. *rhodensis*. Lecithotrophic females, which have oviparous embryos, obtain nutrients from the yolk of the ovum and allocate all nutrient resources to the embryo before fertilization, resulting in larger eggs than matrotrophic females, which allocate resources to offspring throughout gestation (Trexler and DeAngelis, 2003; Buddle et al., 2019). Furthermore, in oviparous animals, egg size does not change significantly after fertilization because the mother no longer supplies nutrients during pregnancy. In viviparous animals, egg enlargement occurs after fertilization, which is associated with nutrient supply from the mother (Wourms, 1981; Huveneers et al., 2011). In this study, the egg and embryo sizes of *A*. *rhodensis* did not change much during embryonic development, although significant differences were observed in the size of the threefold stage embryos. By contrast, significant differences in egg and embryo size were observed among all stages of *T. tufae. T*. *tufae* eggs and embryos at the threefold stage were respectively 2.6- and 3.6-fold larger than at the single-cell stage, suggesting nutrition supply by the mother in the uterus. Notably, removal from the uterus halted embryonic development at the single-cell and lima bean stages in *T. tufae* irrespective of the solution used for incubation. This indicates that the embryos, at least from the single-cell to the lima bean stage, receive nutrients essential for growth from the mother.

Regarding egg permeability, fluorescence was observed only in the region between the eggshell and the embryo in *A*. *rhodensis* (Figure 3). By contrast, all of the fluorescent substances permeated *T*. *tufae* embryos (Figure 3). Olson et al. (2012) reported that in *C*. *elegans*, high-molecular-weight substances did not permeate embryos because of a permeability barrier rather than the eggshell. In this study, ≤70,000 MW molecules penetrated the eggshells of *A*. *rhodensis* and *T*. *tufae*. However, in *A*. *rhodensis*, the permeability barrier surrounding the embryo inhibited this permeation. Indeed, TEM demonstrated that *T*. *tufae* lacks a permeability barrier (Figure 4D), whereas *A*. *rhodensis* does not (Figure 4B). The permeability barrier acts as an osmotic barrier to prevent large molecules or toxins in the external environment from transmit into embryos (Olson et al., 2012). In *T*. *tufae*, the absence of the permeability barrier resulted in increased molecular permeability. Nutrient substances, *e*.*g*., vitellogenin, are typically of high molecular weight. Therefore, the absence of the permeability barrier may be linked to nutrient supply by the mother.

Eggshells have different functions in oviparous and viviparous nematodes. The eggshell of *A*. *rhodensis* was composed of VL, ML, and IL (Figure 4A). By contrast, the eggshell of *T*. *tufae* comprised only a VL (Figure 4C). Our permeability tests indicated that this difference in eggshell structure is not due to increased permeability, as the fluorescent substances passed through the *A. rhodensis* and *T. tufae* eggshells regardless of size. The simplification of eggshell structure is typically associated with the evolution of viviparity (Blackburn, 1993a; Blackburn, 1993b; Heiden et al., 2005). In the eggshell of oviparous animals, a hard layer comprising chitinous or calcareous materials protects the embryos from external stresses. In viviparous animals, the hard layer is absent because there is no need to protect the egg from external stressors. The ML contains chitin and acts as a framework to maintain egg shape (Olson et al., 2012; Stein and Golden, 2018). The absence of the chitin-containing ML likely allows an increase in egg size during embryogenesis. Furthermore, a thinner or absent eggshell promotes gas exchange in the embryo. In reptiles, dissolution or absence of the structure surrounding the embryo, the shell membrane, during pregnancy enhances gas exchange in viviparous taxa (Blackburn, 1998). The absence of the rigid eggshell in *T*. *tufae* may facilitate embryonic gas exchange; this warrants further investigation.

Although the nutrients supplied by the mother to the embryo in *T*. *tufae* are unknown, the developmental changes in structural and functional features of *T*. *tufae* are like those in other viviparous animals. Therefore, *T*. *tufae* is a viviparous rather than an ovoviviparous nematode species. Investigation of the ecology of *T*. *tufae* is required to identify the evolutionary driver of the transition from oviparity to viviparity. Although *T*. *tufae* has only been found in Mono Lake, we recently isolated two other species of *Tokorhabditis* from dung beetles (Ragsdale et al., 2022). Further, *Sudhausia* spp. nematodes sometimes cohabit with *Tokorhabditis* spp. and so are likely to be viviparous (Herrmann et al., 2013; Kanzaki et al., 2017a). Identification of the ecological factors common to these viviparous nematode species may provide insight into the evolutionary forces that drive the transition from oviparity to viviparity. Comparison with the model organism *C*. *elegans* will clarify the evolution of the mechanism of viviparity.

## Data Availability

The original contributions presented in the study are included in the article/supplementary material, further inquiries can be directed to the corresponding author.
